# Social Media as a Sensor of Air Quality and Public Response in China

**DOI:** 10.2196/jmir.3875

**Published:** 2015-03-26

**Authors:** Shiliang Wang, Michael J Paul, Mark Dredze

**Affiliations:** ^1^Johns Hopkins UniversityDepartment of Computer ScienceBaltimore, MDUnited States; ^2^Johns Hopkins UniversityHuman Language Technology Center of ExcellenceBaltimore, MDUnited States

**Keywords:** air pollution, public health surveillance, social media, data mining, text mining, natural language processing

## Abstract

**Background:**

Recent studies have demonstrated the utility of social media data sources for a wide range of public health goals, including disease surveillance, mental health trends, and health perceptions and sentiment. Most such research has focused on English-language social media for the task of disease surveillance.

**Objective:**

We investigated the value of Chinese social media for monitoring air quality trends and related public perceptions and response. The goal was to determine if this data is suitable for learning actionable information about pollution levels and public response.

**Methods:**

We mined a collection of 93 million messages from Sina Weibo, China’s largest microblogging service. We experimented with different filters to identify messages relevant to air quality, based on keyword matching and topic modeling. We evaluated the reliability of the data filters by comparing message volume per city to air particle pollution rates obtained from the Chinese government for 74 cities. Additionally, we performed a qualitative study of the content of pollution-related messages by coding a sample of 170 messages for relevance to air quality, and whether the message included details such as a reactive behavior or a health concern.

**Results:**

The volume of pollution-related messages is highly correlated with particle pollution levels, with Pearson correlation values up to .718 (n=74, *P*<.001). Our qualitative results found that 67.1% (114/170) of messages were relevant to air quality and of those, 78.9% (90/114) were a firsthand report. Of firsthand reports, 28% (32/90) indicated a reactive behavior and 19% (17/90) expressed a health concern. Additionally, 3 messages of 170 requested that action be taken to improve quality.

**Conclusions:**

We have found quantitatively that message volume in Sina Weibo is indicative of true particle pollution levels, and we have found qualitatively that messages contain rich details including perceptions, behaviors, and self-reported health effects. Social media data can augment existing air pollution surveillance data, especially perception and health-related data that traditionally requires expensive surveys or interviews.

## Introduction

Recent studies have demonstrated the utility of social media data sources for a wide range of public health goals. Studies have focused on epidemiological surveillance systems for influenza [1,2] and allergies [3], tracking health behaviors such as smoking [4-6] and exercise [7], identifying mental health trends [8,9], and measuring health perceptions and sentiment [10,11]. These are just some of the many health topics discussed on the social media service Twitter [12], demonstrating the ability of social media to complement traditional public health methods, often providing trends faster than traditional surveillance and insights that are difficult to detect through traditional mechanisms.

However, most work to date has focused on Twitter, emphasizing health topics of major concern in the United States, with little work concerning health issues important in other countries. Only recently has attention been given to studying health in Chinese social media, primarily for the purpose of influenza surveillance [13-16]. Our recent study [17] analyzed the diversity of health content in messages from Sina Weibo (abbreviated as Weibo), a microblogging site popular in China. While many of the health topics were similar to those identified on Twitter (eg, influenza, common cold, exercise, and vision health), some topics were unique to China. Most notably, Chinese social media users often discussed pollution and air quality in China, a major Chinese public health issue [18], which receives less attention on Twitter [19].

Air pollution can have tremendous health consequences, such as increased respiratory and cardiovascular disease [20,21]. Air pollution is a major concern in China, where pollution levels are rising alongside rapid urbanization and industrialization [22,23]. Addressing air pollution requires localized surveillance of pollutant levels. Additionally, it is important to understand public awareness, concern, attitudes, health effects, and behavioral response to air pollution [24]. Researchers have investigated public perceptions of risk regarding pollutants [25], emotional and affective responses to air pollution [26], and behavioral responses to pollution, for example, to understand whether people are taking averting action such as staying indoors [27]. This knowledge is important for guiding public policy efforts to reduce pollution, for informing researchers building accurate models of pollution health effects, and for directing the public on how to best respond and protect themselves. So far, these studies have relied on traditional public health methods, such as surveys, for obtaining necessary data.

In this paper, we investigate whether social media data can be used to identify air quality trends and public response in China. Mining social media offers the potential for these trends to be identified in real time and on a massive scale. We mined Weibo messages for statements about air quality and pollution. We demonstrated two epidemiological uses of these data. First, we compared the volume of air quality messages with fine particle pollution in 74 Chinese cities to evaluate the effectiveness of social media for complementing air quality sensors. Second, we conducted a manual coding analysis of a sample of messages to evaluate the ability for measuring public perception, awareness, and response to pollution, a first step toward quantifying the impact of environmental factors on health.

## Methods

### Data

We collected 93 million messages from Weibo using Weibo’s public API. Starting with a small set of randomly selected seed users, we downloaded the 100 most recent messages from each user, then proceeded recursively to download data for the user’s followers. All messages were collected in December 2013 but the messages were written as far back as 2009. Since we obtained the most recent messages for each user, the bulk are from 2013 (68.42%, 63,789,097/93,225,579). To focus on the health aspect of air quality and pollution, we selected a set of 917,708 messages obtained by filtering using a list of 1282 health-related terms from a Chinese medical dictionary [28], as well as terms added manually, such as terms related to air pollution: pollution (污染), lungs (肺部), and smog (烟雾). These data were originally collected as part of a broad study into health topics in Chinese social media [17]. The text was preprocessed by removing punctuation, common “stop words”, and infrequent words, and performing Chinese word segmentation (see [17] for details).

Weibo requires that users provide city and province upon registration, which is included in the downloaded data. Additionally, each user account has a verified attribute that designates whether it is an individual user (as well as celebrities), a government account, a company account, the media, or others.

To aid additional work on this topic, we are making publicly available the health keywords used to filter Weibo messages, the statistics computed from Weibo for each city and filter, and a list of the Weibo message IDs used in this study along with the filters they matched (see Multimedia Appendix 1). While we are unable to provide the raw Weibo messages per the terms of service, the Weibo public API can be used to directly download messages given the IDs.

### Identifying Air Quality Messages

We experimented with two methods for identifying messages related to air quality or pollution. First, we used a simple keyword-based filter in which we selected messages that contain one of four relevant terms: pollution (污染), air (空气), breathe (呼吸), and cough (咳嗽).

Second, we used Latent Dirichlet Allocation (LDA) [29], a probabilistic topic model, to filter messages that belonged to topics relevant to air quality or pollution. A topic model is a probabilistic model of text data, which has two sets of parameters: each document has a discrete distribution over “topics” and each topic has a discrete distribution over words. When estimating the parameters of this model, the topic-specific word distributions typically give high probability to words that tend to occur together in documents. Each topic can therefore be interpreted as a topically or semantically coherent group of words. These parameters are wholly inferred from a raw text corpus, allowing the model to learn topics specific to data of interest.

**Figure 1:**
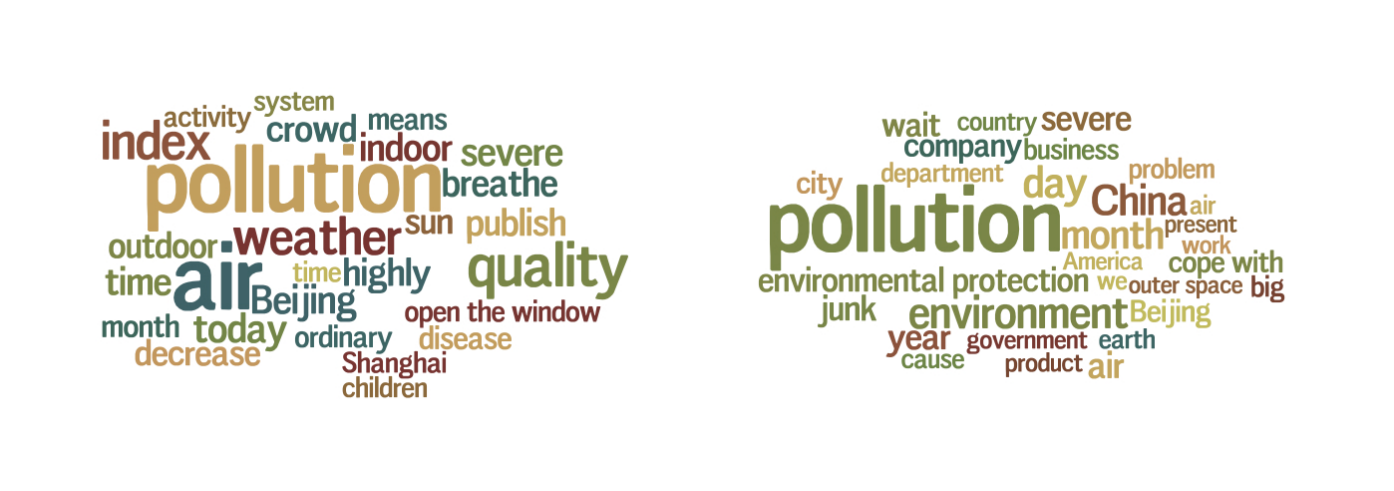
Two pollution-related topics learned from a probabilistic topic model. The left topic is about air quality, and the right topic is about pollution in general.

The LDA model parameters were estimated after 1000 iterations of Gibbs sampling, using 100 topics on our health Weibo dataset. We found two topics whose high-probability words were potentially relevant to air quality, shown in Figure 1 as word clouds. The words in the figure represent the 25 highest-probability words in each topic. Larger words are more probable. The words have been translated from the original Chinese text. The first topic (“AQ”) includes many words related to air quality, while the second topic (“PO”) is more generally about pollution. Since these words are derived from a fully automated method, they contain many words readily recognizable as relevant to the topic, whereas a few are not as clear.

We used these two topics to filter Weibo messages by selecting messages where at least one token was assigned to the given topic by the sampler.

We experimented with combining our two filtering mechanisms—keyword-based and topic model-based filters—by taking their intersection, selecting messages that both contain a particular topic and a particular keyword.

Finally, we experimented with filtering out messages that contained URLs, under the assumption that these messages are likely to be sharing news media rather than personal experiences [30].

### Comparison to Air Quality Measurements

We compared the volume of air quality messages with fine particle pollution (PM2.5) measurements for 74 Chinese cities from 2013. We compared to the average daily value across 2013 as well as the maximum of all daily values. The data came from the State Environmental Protection Department, which began air quality monitoring in 2012 for these 74 cities [31,32]. Fine particles are those less than 2.5 micrometers in diameter, detected with automated monitoring systems that run continuously with at least 85% uptime, beginning August 2013. The sensing methods are described in [33] (Chinese only). We focus on fine particle pollution because it poses a greater health risk than coarse particle pollution [34].

For each of the cities, we computed the volume of social media activity as the number of messages from the city after filtering for relevance, divided by the total number of messages from the city across the entire dataset. This normalization technique has been previously used for obtaining rates from Twitter data [2]. We measured the Pearson correlation (n=74) between the Weibo volumes and the city PM2.5 values.

### Analysis of Message Content

We coded 170 randomly selected messages. We labeled whether the message discussed air quality or air pollution, and if so whether it described a firsthand experience by the user (rather than a general awareness), and if so whether the user reported a change in behavior (eg, wearing a mask), and whether the user expressed concern for his or her health. If a message discussed air quality, we also labeled whether the user requested that action (eg, by the government or community) be taken to improve air quality.

Of messages expressing a health concern, we noted any specific symptoms or health conditions explicitly identified in the message that were perceived to be a result of poor air quality.

Messages were coded independently by two annotators and disagreements were resolved after discussion with a third annotator. We measured the agreement between the two primary annotators using Cohen’s kappa score.

### Message Classification

Finally, we experimented with a supervised machine learning approach for identifying relevant messages, using the 170 coded messages as training data. While the messages were not coded for the purpose of training a model, this is a natural experiment to try because messages were labeled with details about relevance.

We used a cascade approach similar to that of Lamb et al for influenza in social media [30], first classifying messages for relevance to air quality, and then classifying messages indicating a firsthand experience (rather than a more general awareness). The first classifier (relevance) was trained on all 170 messages, while the second classifier (firsthand experience) was trained on the subset of messages labeled as relevant. The two classifiers were constructed as logistic regression models using 1-, 2-, and 3-gram word features.

We applied the classifiers to the full set of messages and, as with the other filters, we measured the correlation between the volume of messages identified by the classifiers with the government data.

## Results

### Data Statistics

Of the 917,708 messages that were filtered for all health-related keywords, 405,467 messages came from the 74 cities with PM2.5 data, with an average of 5479 messages per city (median 3079).

Almost all user accounts, 99.31% (432,862/435,873), were considered “individual” users (not government, business, or media). Government accounts were 0.14% (613/435,873) of users, 0.49% (2147/435,873) were companies, and 0.06% (251/435,873) were media accounts. Thus our data represents individual users as opposed to organizations or governments.

In total, regardless of location, the four keywords matched 75,912 messages, the AQ topic matched 15,763 messages, and the PO topic matched 45,172. For the air quality comparison, we filtered these messages based on the 74 available cities, while the analysis of message content drew from the total dataset.

### Comparison to Air Quality Measurements

**Figure 2:**
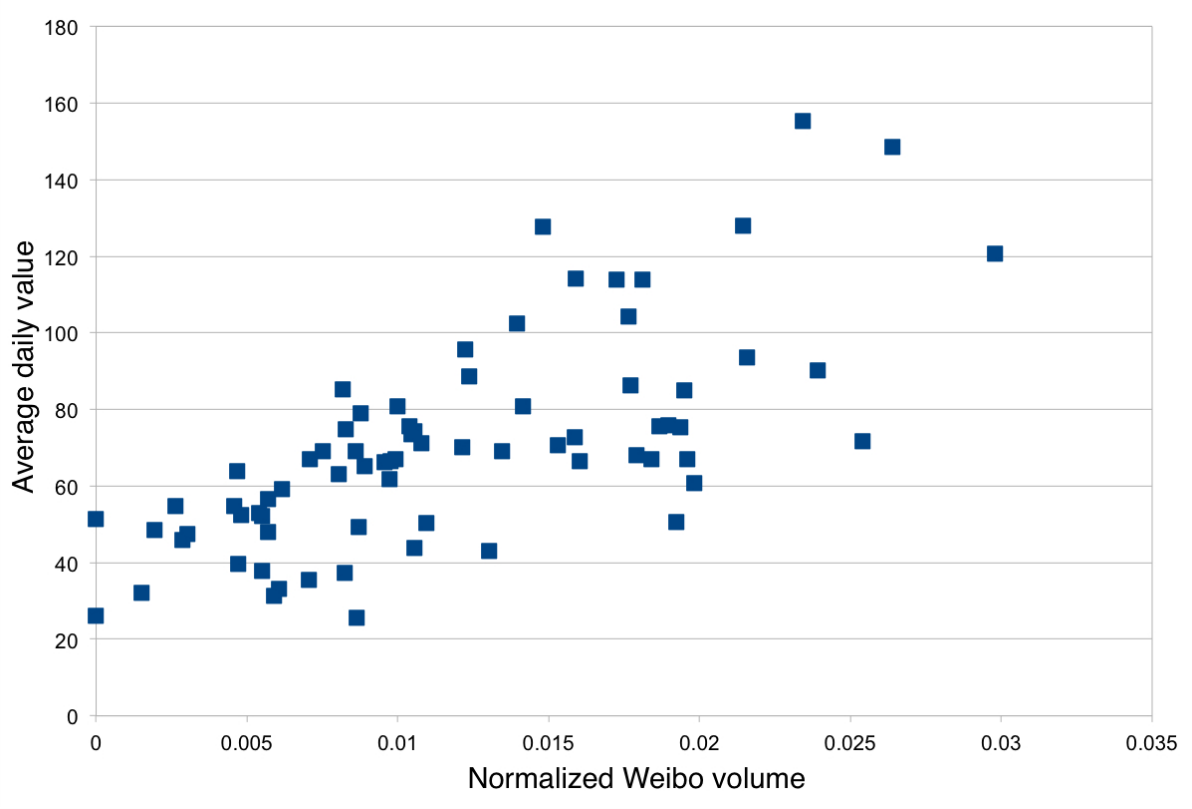
Scatter plot showing average daily PM2.5 values (y-axis) and the Weibo rate for 74 cities using our most correlated filter, AQ+”pollution” (r=.703).

Table 1 shows the correlations between the volume of filtered messages in each of the 74 cities and the PM2.5 values. None of the differences between correlations when using the maximum daily value (MDV) versus the average daily value (ADV) are statistically significant, but the highest correlations are with ADV. Figure 2 shows a scatter plot of these values for our best filter.

Of the individual keyword filters, “air” has the highest correlations, while of the topic model filters, the AQ topic correlates best. Additionally, we discovered that the correlations can be improved further by combining the best topic model (AQ) with the best keywords (“air” and “pollution”). Combining the AQ topic with “pollution” yields the highest correlation.

If we exclude messages that contain URLs, the correlations improve in all cases except with the PO topic filter.

The highest correlation achieved is with the AQ+”pollution” filter on messages without URLs, at .703 (*P*<.001).

**Table 1:** Correlation of messages matching each filter in 74 cities to the average (ADV) and maximum (MDV) daily PM2.5 values in 2013.

Filter	Including URLs			Without URLs		
	Number of messages	Corr. (ADV)^a^	Corr. (MDV)^b^	Number of messages	Corr. (ADV)	Corr. (MDV)
AQ^c^ topic	7665	.546	.545	5866	.583	.565
PO^d^ topic	21,902	.361	.421	17,696	.286	.387
“air”	6321	.552	.593	4949	.610	.637
“pollution”	15,809	.458	.474	12,044	.606	.633
“breathe”	4807	.351	.257	4454	.361	.290
“cough”	12,437	−.005	−.151	11,921	.027	−.023
AQ+“air”	4133	.564	.557	3103	.623	.579
AQ+“pollution”	4866	.630	.619	3766	.703	.657

^a^Corr. (ADV): Correlation, average daily value

^b^Corr. (MDV): Correlation, maximum daily value

^c^AQ: air quality

^d^PO: pollution

### Analysis of Message Content

We analyzed 170 messages from the AQ topic, which had the highest correlation of the two topics. We did not filter for specific keywords so that we could get a broader set of messages. To target messages that were more strongly relevant to the topic, we selected messages such that the message’s topic distribution assigned more than a 0.1 probability of the document being about this topic, which yields messages with at least two tokens of this topic on average.

**Figure 3:**
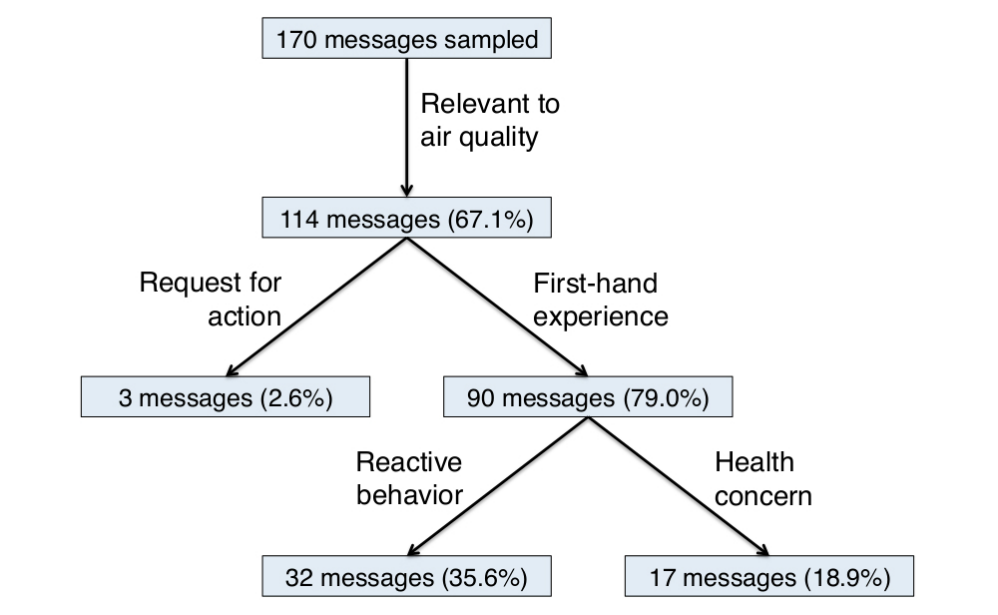
Summary of annotation results on sample of 170 messages. Tree structure indicates which codes are dependent on their parent codes. Different branches are not mutually exclusive.

These results are summarized in Figure 3. We found that 114 (67.1%) messages sampled through this filter were actually relevant to air quality or air pollution. Of those 114 messages, 90 indicated a firsthand experience (79.0%). Of those 90 messages, 32 (36%) mentioned a reactive behavior, and 17 (19%) expressed a concern for the user’s health.

Three (2.6%) out of 114 relevant messages requested that action by taken to improve the air quality. One message declared a need to reduce carbon emissions, while the other two more generally called for cleaner air (one was directed at the government).

The most common reactive behavior was wearing a face mask, while other behaviors include washing clothes and staying indoors.

Of the 17 messages expressing a health concern, five reported a cough, three reported a sore throat, and two reported dry or peeling skin. Various health conditions were also reported: rhinitis (four messages), allergic rhinitis (one), pharyngitis (one), and asthma (one).

A common pattern that we noticed in firsthand messages that did not belong to the more specific categories (reactive behavior or health concern) is the expression of emotions such as anger or sadness; however, we chose not to quantify this characteristic because it is difficult to define concretely.

Table 2 shows annotator agreement scores from the initial annotations, before disagreements were resolved. Annotator agreement percentages ranged from 78% to 97%. There was very high agreement on whether messages were relevant to air quality, whether the user requested action to improve quality, and whether the user expressed a reactive behavior.

There was less high agreement about whether messages were a firsthand experience, which was sometimes ambiguous and difficult to determine. The lowest agreement was on whether the user expressed a health concern. Annotator divergence primarily stemmed from disagreement over whether a general discomfort should be classified as a health concern. For example, many users expressed discomfort breathing and thus wore a mask. After discussion, we did not count such messages as health concerns, unless health concerns were explicitly stated. Table 3 shows examples of messages that illustrate the various annotations.

**Table 2:** Percentage of annotated messages matching the criteria, along with annotator agreement statistics for each question.

Code	Agreement, n (%)	Agreement (kappa)
Relevant to air quality, n=170	160 (94.1)	.869
Request for action, n=107	104 (97.2)	.557
Firsthand experience, n=107	87 (81.3)	.363
Reactive behavior, n=78	73 (93.6)	.864
Health concern, n=78	61 (78.2)	.429

**Table 3:** Examples of messages with various labels (the original Chinese Weibo is shown, followed by an English translation).

Label	Message
Not about pollution	累昏厥了。牢笼一般的机场巴士, 传说中根本不叫花钱的物价, 空气里的尿骚味以及灰蒙蒙的天。无论哪顿饭除了咖喱还是咖喱。 I was tired and fainting. The high price, the urine-scented air, and the heavy, gray day made the airport bus feel like a cage. Plus, every meal on the airport bus was curry.
About pollution, not a firsthand experience	老外说: 这幅画表达的是污染程度的北京。PM爆表。 A foreigner said that this picture shows the serious pollution of Beijing. The PM value is too high.
Request for action	不能在空气质量重度污染时才想起低碳行动! Don’t wait until the air has already been heavily polluted to start reducing carbon.
Firsthand, reactive behavior	今晚想出去跑步,一查空气指数,还是轻度污染,在家避毒吧。 I want to go running this evening. However, it is lightly polluted based on the air pollution index, so I have to stay at home.
Firsthand, health concern (+ reactive behavior)	三天前开始咳嗽。一定是北京污染的天气有关, 以后出门戴口罩[生病]。 I start coughing three days ago. It must be caused by the pollution in Beijing! I will wear a mask when I go outside [sick].

### Message Classification

We evaluated the classifiers with 10-fold cross validation. The first classifier, trained on 170 messages with 114 positive for relevance, achieved a cross-validation accuracy of .794 (precision .794, recall .947). The second classifier, trained on 114 messages with 90 positive for firsthand experience, achieved a cross-validation accuracy of .718 (precision .689, recall .867).

Because these classifiers were trained on messages that were already filtered by the AQ topic, we then applied the classifiers to the subset of messages containing the AQ topic, similar to the AQ+“pollution” filter. Using this filter, the correlations with pollution data are .718 (ADV) and .664 (MDV). These are both higher than the best correlation reported in Table 1, though not by significant margins.

## Discussion

### Principal Findings

It is encouraging that even simple content filtering produced moderately high correlations with existing surveillance data. This suggests that lightweight methods can be used for social media-based air quality monitoring. We also showed that better text modeling, through topic modeling and supervised classification, can further improve the correlations. It is perhaps surprising that the supervised classifier did not greatly improve the correlation over simply combining the “pollution” keyword filter with the AQ topic. This may be because the training set of 170 messages was small. In a recent study concurrent with our own, Mei et al found machine learning to help identify air pollution trends in social media [35].

Some of the keyword filters did quite poorly. While “cough” and “breathe” are related to air quality, they are related to much more popular topics as well, yielding filters with low specificity. This demonstrates the benefit of basic natural language processing via topic models. Topic models, which make probabilistic inferences about the topic composition of a message, led to improved correlations when combined with the keyword filters. These models make use of the entire context of a message, which can provide a better relevance model than individual words or phrases. Topic models can also introduce noise, since the models are unsupervised, which we believe is why combining the topic model with a highly relevant keyword like “pollution” correlates better than either filter alone.

Another point to consider is that our filters identify whether a message is *about* air quality, but not *what* the quality is. A promising research direction is to infer a scalar value of air quality based on message content. Natural language processing techniques used for sentiment analysis—the task of quantifying the degree to which text expresses a positive or negative sentiment [36]—could perhaps apply here. For example, certain words like “terrible” or “worst” might indicate worse air quality than simply “bad”.

Additionally, we hypothesize that there is a potentially much larger number of messages that could be mined. Extrapolating from a 67% relevance rate of 170 out of 15,763 messages, we estimate there are at least 10,000 messages about air quality in our crawled dataset. Additionally, our entire dataset contains only 93 million messages, a much smaller dataset than those typically used in Twitter research today; for example, Paul and Dredze [3] used a general collection of 2 billion messages to study health topics on Twitter. Since Weibo has more registered users than Twitter, we expect data collection targeted at obtaining air quality messages would obtain a much larger collection.

Our coding results suggest a promising direction in using Weibo messages to understand health concerns, behavioral responses, and health impacts of environmental factors. We found users reporting on all three. While previous work suggests that users will report on well-being during an air pollution crisis [37], we are the first to show that Chinese users make relevant statements on social media services. By building systems that automatically identify these three issues and aggregate them over many users, we could greatly expand traditional surveillance capabilities and inform health interventions.

We believe social media-derived information will be especially advantageous for measuring public perception and response. This is information that cannot be captured with physical sensors, and instead relies traditionally on surveys, panels, and interviews. However, measuring the perceived level of pollution can be just as important as the objective level, as the perceived level is a stronger predictor of willingness to reduce pollution [26]. In this sense, social media reports are more akin to citizen complaints than physical sensors. Although not objective, citizen complaints can complement physical surveillance, and complaints often result in follow-up investigations by regulators in China [38]. Formal complaints likely have different characteristics than the informal complaints found in social media. Social media complaints tend to be general, and the threshold of perceived pollution before writing a complaint is quite likely lower in social media. As social media becomes a more common outlet for citizen complaints, the relationship between these systems of complaint will be important to understand [39].

As with perception, behavioral response to pollution is hard to measure, and often is not measured at all, but rather inferred or assumed [27]. There is therefore a clear knowledge gap that social media data can help fill. A type of behavioral response of particular interest is response to public awareness campaigns or health advisories regarding pollution [40]. Because awareness campaigns and advisories may take place over a short period of time—in some cases, just a single day—it can be difficult to measure their outcome. This has motivated researchers to use Web-derived data to measure the effectiveness of such campaigns, for example using Web search activity to understand World Tobacco Day [41] and Breast Cancer Awareness Month [42]. Having shown that social media users report their perceptions and behaviors regarding air quality, we believe that this data could similarly be used to understand the effectiveness of pollution advisories. This falls into a broader trend of using digital data to support research in behavioral medicine [43].

Finally, we found that many users report perceived health effects of pollution, including specific conditions such as asthma and symptoms such as cough. Previous research has shown that self-reports of health status can be combined with reports of air pollution exposure to understand the associated health effects [44,45]. Social media data, including our Weibo collection, offer passive self-reporting at a much larger scale than what can be collected through traditional, active methods, such as interviews. Such data can augment our understanding of environmental health effects, especially because social media reports include people who experience symptoms but do not seek care, and thus fall outside of what is captured in medical records. Furthermore, research on the health effects of pollution often focuses on more serious outcomes such as disease, while social media reports contain evidence of milder but still important effects, such as discomfort and irritability [24]. Indeed, reports of discomfort were so common in our dataset that we decided to exclude them from consideration as a “health concern” in our coding analysis, yet there is clearly potential for the data to help quantify these effects.

An important limitation to consider is the effect of government censorship on using social media for informatics in China. Studies have shown that collective action and mentions of certain politically sensitive topics are subject to censorship [46-48], but it is not clear whether this would affect pollution-related media. More research is required on this topic, but it is clear from our analysis that many messages describing experiences with air quality can be found in this data source.

### Conclusions

To conclude, our findings show that social media messages in China contain a variety of relevant firsthand user reports of air quality, and the volume of these messages correlates with air particle pollution levels in 74 Chinese cities. This was a proof of concept study. Our goals were to understand the content of air quality-related messages, through a qualitative coding of a sample of messages, and to validate the relevance of the messages, by correlating the social media data with existing surveillance data. Our results validate both the quality of these messages and suggest that mining their content can deliver important epidemiological insights into environment health.

## Multimedia Appendix 1

Health keywords used to filter Weibo messages, statistics computed from Weibo for each city and filter, and list of Weibo message ids used along with the filters they matched.

## Abbreviations

ADV: average daily value
AQ: air quality
LDA: Latent Dirichlet Allocation
MDV: maximum daily value
PO: pollution
